# Use of Learning Analytics Data in Health Care–Related Educational Disciplines: Systematic Review

**DOI:** 10.2196/11241

**Published:** 2019-02-13

**Authors:** Albert KM Chan, Michael G Botelho, Otto LT Lam

**Affiliations:** 1 Prosthodontics Faculty of Dentistry The University of Hong Kong Sai Ying Pun China (Hong Kong)

**Keywords:** education, e-learning, learning analytics, learning management systems, online learning, systematic review

## Abstract

**Background:**

While the application of learning analytics in tertiary education has received increasing attention in recent years, a much smaller number have explored its use in health care-related educational studies.

**Objective:**

This systematic review aims to examine the use of e-learning analytics data in health care studies with regards to how the analytics is reported and if there is a relationship between e-learning analytics and learning outcomes.

**Methods:**

We performed comprehensive searches of papers from 4 electronic databases (MEDLINE, EBSCOhost, Web of Science, and ERIC) to identify relevant papers. Qualitative studies were excluded from this review. Papers were screened by 2 independent reviewers. We selected qualified studies for further investigation.

**Results:**

A total of 537 papers were screened, and 19 papers were identified. With regards to analytics undertaken, 11 studies reported the number of connections and time spent on e-learning. Learning outcome measures were defined by summative final assessment marks or grades. In addition, significant statistical results of the relationships between e-learning usage and learning outcomes were reported in 12 of the identified papers. In general, students who engaged more in e-learning resources would get better academic attainments. However, 2 papers reported otherwise with better performing students consuming less e-learning videos. A total of 14 papers utilized satisfaction questionnaires for students, and all were positive in their attitude toward e-learning. Furthermore, 6 of 19 papers reported descriptive statistics only, with no statistical analysis.

**Conclusions:**

The nature of e-learning activities reported in this review was varied and not detailed well. In addition, there appeared to be inadequate reporting of learning analytics data observed in over half of the selected papers with regards to definitions and lack of detailed information of what the analytic was recording. Although learning analytics data capture is popular, a lack of detail is apparent with regards to the capturing of meaningful and comparable data. In particular, most analytics record access to a management system or particular e-learning materials, which may not necessarily detail meaningful learning time or interaction. Hence, learning analytics data should be designed to record the time spent on learning and focus on key learning activities. Finally, recommendations are made for future studies.

## Introduction

Learning analytics has been defined [1] as “the measurement, collection, analysis and reporting of data about learners and their contexts, for purposes of understanding and optimizing learning and the environments in which it occurs”; this broad definition allows the inclusion of virtually anything related to learning. From a more holistic perspective, Picciano [2] proposed that learning analytics is a process that can provide conclusions for decision making through the examination of data such as for helping colleges and universities to identify and evaluate strategies for improving the retention of students. In addition, it can help instructors to decide if an intervention is needed to assist students.

More recently, the 2016 Horizon Report [3] increasingly emphasized the usage of Web-based tools and platforms and described learning analytics as “an educational application of web analytics aimed at learner profiling, a process of gathering and analyzing details of individual student interactions in online learning activities.”

While a universal definition of learning analytics has not yet reached a consensus, there is general agreement that it is a relatively new [4] and emerging [5,6] tool in academic research, which can be used to track and store students’ Web-based learning activities [5,7]. Higher education institutions collect a vast amount of information from students regarding their use of e-learning resources in the form of activity logs and other digital footprints such as time and date, student demographics, course enrollments, survey questionnaires, library usage, and academic grades [8]. A wide range of e-learning resources ranging from administration, assessment, assignment, quiz, multimedia, to collaboration, to name a few, now can be found integrated into learning management systems (LMS). Looking at such learning analytics data can allow researchers to investigate and examine relations between students’ e-learning use and academic performance [9].

Furthermore, learning analytics offers a convenient and potentially accurate method to capture students’ interactions with the e-learning resources, which was not achievable in the past. Previously, student engagement was measured by class attendance [10] and self-reported questionnaires. For example, questions such as “How frequently do you use *X* ” [11] would be administered to acquire information from students about their e-learning usage. However, self-reported answers have the disadvantage of being inaccurate (recall bias). With learning analytics, investigators can collect information such as the exact number of video watches, and time and date the videos were viewed.

One of the main practical applications for learning analytics data is the investigation of students’ e-learning usage and the examination of its effect on their academic performance. Students’ e-learning usage behaviors, such as the number of log-ins, time spent on e-learning platforms, and use of other resources, have been studied and found to be positively associated with academic performance outcomes such as summative multiple-choice question (MCQ) exam scores [12,13]. In addition, learning analytics can allow educators to examine an individual student’s tracked Web-based activities and search for any at-risk students with one of its predictive functions, then intervene by providing feedback and instructional content [5,14,15].

Although learning analytics applications in higher education have received increasing attention in recent years [5], a limited number of studies have investigated its use in health care-related educational studies. With the acknowledgment of advantages in learning analytics data, this paper aims to review the use of learning analytics in e-learning in health care educational studies with regards to how it is reported and how this may be related to learning outcomes.

## Methods

Textboxes 1 and 2 present details pertaining to study populations, interventions, comparisons, and outcomes are presented in accordance with the PICO (population, intervention, comparison, and outcome) model [16]. Textbox 3 details the keywords used in the systematic search of 4 electronic databases (MEDLINE, EBSCOhost, Web of Science, and ERIC). The end search date was August 25, 2017.

We limited the searches to papers that spanned from 2000 to 2017, were involved in tertiary-level education in health care-related disciplines, and were in English. We excluded gray literature. In addition, reviews and commentary columns were discarded, and multiple papers on the same research data were excluded.

After deleting duplicates, all papers retrieved from these initial search criteria were subjected to a screening process by reading titles and abstracts. The detailed information regarding the data type, e-learning content, learning outcome, and key findings were analyzed.

**The inclusion criteria based on the PICO (population, intervention, comparison, and outcome) model.**

P: Studies that involved undergraduate or postgraduate students in health care-related disciplines (eg, dentistry, medicine, nursing, and pharmacy).

I: Studies that explored learning analytics were included. Owing to the fact that some studies reported learning analytics data but did not specifically utilize the term “learning analytics,” this review also included studies that used learning management systems, educational technologies, or other tools that contained digital footprints of students’ e-learning usage.

C: Regarding comparisons, Jin and Bridges [17] suggested that experimental designs should not be considered exclusively because a large proportion of educational research in these fields is case-based as well.

O: Studies that mentioned quantitative measurements of learning outcomes, such as student academic performance related to their knowledge or skill assessment were included.

**The exclusion criteria based on the PICO (population, intervention, comparison, and outcome) model.**

P: Studies that reported only instructors, staff, or physicians were excluded.

I: Anything other than those included in the inclusion criteria.

C: Studies that involved only qualitative methods were not included.

O: Anything that did not mention the word “learning outcome” or “GPA/grade”

**The database search strategy.**

(clinical OR dent* OR med* OR nursing OR pharmacy) AND

(undergraduate OR postgraduate) AND

(academic achievement OR academic attainment OR assessment OR GPA or grade or consumption) AND

(educational technologies OR learning management system OR content management system OR virtual learning environment OR technology enhanced learning OR learning analytics OR digital footprint OR e-learning OR logs) AND

(education OR learning OR training)

## Results

### Principal Results

**Figure 1:**
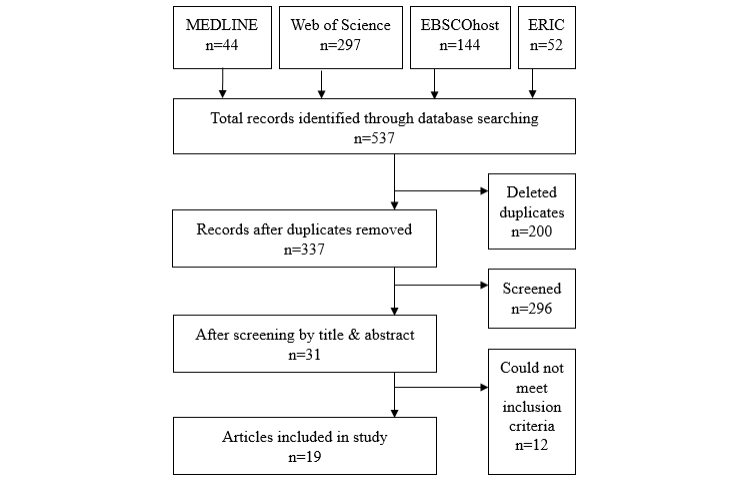
Flowchart of the search process.

The search of the 4 databases resulted in 537 papers (Figure 1), and a total of 337 papers were obtained after the removal of duplicate results. These were further screened by the same independent researchers, and 296 papers were excluded because of not satisfying the inclusion criteria. Based on the content in abstracts, full texts of 31 potentially effective papers were retrieved and screened, from which a total of 19 papers met all the inclusion criteria and were subjected to content evaluation.

### Learning Analytics Data Types

A total of 19 studies reported analytic data based on the number of connections, time spent, or combinations of these and other analytic approaches such as the number of forum posts, MCQ exam scores, and responses of perception questionnaires (ie, satisfaction). Table 1 summarizes the types of learning analytics data among the studies.

**Table 1:** The list of learning analytics data type.

Paper	Learning analytics data type				
	Number of connections	Time spent	Both	Number of posts	Multiple-choice question or Likert
Boye et al [18]		✓			
Catteau et al [19]			✓		✓
Chastonay et al [20]	✓			✓	
Colthorpe et al [21]	✓				
Costa-Santos et al [22]	✓				
Critchley et al [12]			✓		✓
Davidson and Candy [23]		✓			✓
DiLullo et al [24]	✓				
Franson et al [25]			✓		
Garrett et al [26]			✓		
Gurpinar et al [27]	✓				
Kleinsorgen et al [28]			✓		✓
Kukolja-Taradi et al [29]	✓			✓	✓
Lameris et al [30]			✓		
Mahnken et al [31]			✓		
Poot et al [13]	✓				
Reimer et al [32]			✓		
Romanov and Nevgi [33]			✓	✓	✓
Saqr et al [34]			✓	✓	✓

Of the 19 studies, 7 recorded the number of connections (ie, log-in or click or hit or visit or access, etc) to the learning platforms [13,20-22,24,27,29]. Of these, 3 studies only recorded connections to the platform or folder and not the specific learning items contained within [20,21,24].

Two studies reported only the length of time students spent on certain e-learning activities such as e-learning animations [18] and game-based platforms [23]. However, 10 studies used both the number of connections and length of time spent on the e-learning activity by students [12,19,25,26,28,30-34] (eg, the number of connections to the e-platform and the accumulative time spent completing the Web-based modules [19], or connections and time on pages of an e-portfolio system [26]).

### The Nature of E-Learning Content

The types of e-learning interventions varied widely (Table 2), with the more commonly used e-learning formats including videos and animation clips [12,18,19,21,23,24,28,29,32,33], Web-based text documents (eg, handouts, book chapters, journal papers, and slides) [12,19,20,22,24-29], Web-based tests or quizzes [19,22,27-33], and URL links to external Web-based resources [22,27-29].

In particular, 10 papers reported the use of videos, which ranged from animations and lecture recordings, to laboratory precaution clips. In addition, 10 papers utilized e-learning content presented in the form of Web-based text, including but not limited to, slides, PDFs, text documents, and scanned book chapters. A total of 10 studies investigated the use of e-assessments (eg, tests and quizzes), and 4 studies used URL links to external websites. The majority (14/19) of the studies used >1 type of e-learning resource, and 5 studies used a single type [13,18,21,30,31].

### Outcome Measures—Learning and Evaluation

The learning outcomes investigated in the studies were limited to examination or test results with outcomes reported as either a single exam score or a final course grade, which could be a combination of midsemester exams, written assignments, and the end-of-semester exam results. Overall, 14 papers documented learning outcomes (Table 3) as measured by either an end-of-course exam [18,25,27,29,30,32,33] or a combination with course assessments [12,13,21-23,31,34].

**Table 2:** The list of e-learning content.

Paper	e-Learning content				
	Video	Text doc	Test or quiz	URL	Others
Boye et al [18]	✓				
Catteau et al [19]	✓	✓	✓		✓
Chastonay et al [20]		✓			✓
Colthorpe et al [21]	✓				
Costa-Santos et al [22]		✓	✓	✓	✓
Critchley et al [12]	✓	✓			✓
Davidson and Candy [23]	✓				✓
DiLullo et al [24]	✓	✓			✓
Franson et al [25]		✓			✓
Garrett et al [26]		✓			✓
Gurpinar et al [27]		✓	✓	✓	✓
Kleinsorgen et al [28]	✓	✓	✓	✓	✓
Kukolja-Taradi et al [29]	✓	✓	✓	✓	✓
Lameris et al [30]			✓		
Mahnken et al [31]			✓		
Poot et al [13]					✓
Reimer et al [32]	✓		✓		
Romanov and Nevgi [33]	✓		✓		✓
Saqr et al [34]			✓		✓

Five studies did not include objective learning outcomes [19,20,24,26,28]; of these, 1 reported self-efficacy perception from students [19], while another paper mentioned it did monitor assessments and students completed exams [20] but did not subject these data to any statistical analysis nor descriptive statistics. The remaining 4 papers did not include any learning outcome variables in the study designs.

In this study, 14 papers reported student feedback or evaluation questionnaires (Table 3), which assessed their satisfaction toward the e-learning resources [12,13,18-20,22-24,26-30,32] and were all positive. For example, Boye et al [18] reported that the e-learning was well appreciated by students. Garrett et al [26] reported students valued the accessibility and convenience that came from an electronic portfolio. A total of 9 studies incorporated both objective learning outcomes and evaluation questionnaire data [12,13,18,22,23,27,29,30,32].

### Statistical Analyses

Of 19 papers, 5 reported only descriptive statistics [20,23,24,26,32]. The remaining 14 studies [12,13,18,19,21, 22,25,27-31,33,34] performed additional statistical analyses, including the Mann-Whitney *U*-test [18,21,27,33], Student *t* test [21,27,31,33], regression (linear, multiple, or logistic) analyses [12,18,25,30,34], analysis of variance (univariate analysis of variance and multivariate analysis of variance) [13,22,30,31], and correlation tests (Pearson and Spearman) [19,31,34].

### Relating Learning Analytics Data to Learning Outcomes

With regards to the relationship of learning analytics data to objective learning outcomes, 12 of 13 studies demonstrated significant results [12,13,18,19,21,22,25,29-31,33,34], as presented below. One study [19] only analyzed students’ learning analytics on their self-efficacy responses as a replacement of learning outcome.

#### Within the Cohort Relationship of Learning Outcomes and Learning Analytics Data

Perhaps, the most meaningful examination of learning analytics is how it relates to learning outcomes. In a study examining radiology students’ consumption of “e-cases,” a significant correlation was found between their “improvement in knowledge” (pre- and postcourse assessments) and the number of e-cases accessed for those who chose to access e-cases (*r*=0.50, *P*=.003) and those who were required to access (*r*=0.46, *P*=.008) [31].

In a blended learning course for “introductory medical education,” the first-year medical students’ frequency of log-ins to access the LMS was found to have the strongest correlation with their final grade (*r*=0.47, *P*<.01). The second strongest correlation to the final grade was the number of attempts that students took the formative quiz (*r*=0.46, *P*<.01), and the third was students’ formative assessment grades (*r*=0.43, *P*<.01) [34].

**Table 3:** Reported learning outcomes, self-evaluations, and statistical tests.

Paper	Learning outcome (final exam or grade)	Evaluation (satisfaction)	Statistical test (eg, learning outcome versus e-learning)
Boye et al [18]	✓	✓	✓
Catteau et al [19]	Self-efficacy	✓	✓
Chastonay et al [20]		✓	Descriptive
Colthorpe et al [21]	✓		✓
Costa-Santos et al [22]	✓	✓	✓
Critchley et al [12]	✓	✓	✓
Davidson and Candy [23]	✓	✓	Descriptive
DiLullo et al [24]		✓	Descriptive
Franson et al [25]	✓		✓
Garrett et al [26]		✓	Descriptive
Gurpinar et al [27]	✓	✓	✓
Kleinsorgen et al [28]		✓	✓
Kukolja-Taradi et al [29]	✓	✓	✓
Lameris et al [30]	✓	✓	✓
Mahnken et al [31]	✓		✓
Poot et al [13]	✓	✓	✓
Reimer et al [32]	✓	✓	Descriptive
Romanov and Nevgi [33]	✓		✓
Saqr et al [34]	✓		✓

In a study in which case-based e-learning scenarios were used to assist medical students in learning anesthesia diagnostic decision making, learning analytics data, such as the number of students’ completed e-cases, were recorded in an LMS. It was found that students’ marks in the final MCQ test (*r*=0.21, *P*<.05), as well as 2 graded case reports (*r*=0.25, *P*<.01 and *r*=0.30, *P*<.01), were significantly correlated to their second attempt in completing the e-cases and the number of log-ins they made to the LMS (MCQ: *r*=0.18, *P*<.05; case report 1: *r*=0.24, *P*<.01; and case report 2: *r*=0.32, *P*<.01) [12].

In a study concerning a “Teaching Resource Centre (TRC) database” for clinical pharmacology, researchers performed a regression analysis and found that students in 2 separate academic years could increase their grades by 32% (*P*<.001) and 55% (*P*<.001) with more time spent on accessing the “database” [25].

Similarly, Boye et al [18] found that students with intermediate scores in their immunology tests improved their outcome by 3.6% for every hour of watching animated e-learning clips (*P*=.005) from regression analysis to their learning analytics data.

#### Within the Cohort Comparison of Learning Outcomes and Learning Analytics Data

Most studies in this review were interested in finding differences in learning outcomes from different groups of students according to their learning analytics records. Researchers would group students according to the e-learning usage intensity and looked for between-group differences. In a study [21] examining video consumption by physiotherapy or speech pathology students, a marked difference was observed between the groups of high and low “performers.” The high or low performer categorization was defined by considering a range of students’ access to lecture recordings, “meta-learning” tasks, student submission dates of assignments, and assignment and course grades through cluster analysis. Perhaps counterintuitively, students who “performed” better academically were found to have watched lesser Web-based lecture video recordings than students who performed poorer (*P*<.05). Furthermore, the paper reported that higher “performers” achieved a higher course grade than lower “performers” (*P*<.001).

Costa-Santos et al [22] stratified medical students’ Web-based “mini-test” grade results as low, medium, and high and found significantly different outcomes between these on their final exam grades in the biostatistics (*P*<.001) and medical informatics (*P*<.001) modules. Furthermore, they observed that the average grades on the final exam were higher when the “mini-test” results were higher.

In a Web-based course about “acid-base balance in humans,” it was reported that medical students’ “knowledge gain” was significantly improved (*P*<.001) by the e-course [29]; this was determined by students’ “knowledge gain” based on pre- and posttest scores. Furthermore, frequencies of “students’ logs” and students’ visited pages were recorded; however, no statistical analysis was performed relating to the learning outcomes.

A study examining completed modules on an internet-based app found that medical students classified as moderate (*P*=.04) and intensive users (*P*<.001) scored significantly higher than nonusers on the final exam [30]. Each module contained only MCQs, and the study aimed to determine whether a formative testing approach would be effective in stimulating students’ study performance. The classification of nonuser or moderate or intensive was defined by counting the number of modules completed.

In a physiology course where students created and answered peer-generated questions, students who logged into the platform showed significantly higher scores in a summative test compared with those who did not log on to the platform (*P*=.001) [13]. While the number of questions created and answered was recorded, these were not tested for their association with summative test scores.

In another study, medical informatics students who watched Web-based videos had significantly higher final exam scores when compared with nonvideo-watchers (*P*=.007) [33]. Nevertheless, the frequencies of video watches were documented from students’ self-administered questionnaires and were not recorded digitally.

While most papers had shown significant results, Gurpinar et al [27] and Mahnken et al [31] reported otherwise. Gurpinar et al [27] created a website with several pages that contained various e-learning resources. The frequencies of page visits were recorded and it was found that medical students who visited the pages during problem-based learning period exhibited slightly higher exam marks than those who did not visit any pages; however, the result was not statistically significant (*P*=.12).

Mahnken et al [31] did not find a significant difference (*P*=.54) in “improvement in knowledge” (pre- and postcourse assessments) between radiology students who viewed e-cases and those who did not; this is despite the finding that within the stratum of e-case consumers, marked correlations with “improvement in knowledge” were found for both groups of students who were required and not forced to consume the e-cases.

## Discussion

### Principal Findings

Prior educational studies utilizing learning analytics data in nonhealth care disciplines have reported improvements in learning outcomes with increased e-learning interactions [35-37]; these studies have supported the use of e-learning materials as effective in improving students’ academic performance. The findings are congruent with the data from the health care disciplines.

The majority of papers reviewed in this study utilized >1 type of e-learning material, with videos, Web-based documents, and quizzes being the 3 most common; this reflects a diversity of needs and functionalities among course designers in health care curricula. For example, showing a video can provide easier understanding than a description in the text of a complex concept or clinical procedure. Conversely, presenting a written document may be more concise than other means of multimedia e-learning content. Woodham et al [38] found that students preferred a text-based format and believed that the use of video slowed their pace to review and appraise the learning materials in their problem-based learning curriculum. As a result, providing the required content appropriately and efficiently for students’ use may increase their engagement in e-learning use. Based on the diverse formats of e-learning (ie, video, PDF, quiz, etc) and the combinations of these used, the most effective e-learning formats to support learning has not been determined. Hence, further research is needed.

While the majority of studies found that assessed learning outcomes were improved by using e-learning resources, 2 papers reported insignificant findings but did not explain why this occurred [27,31]. A possible reason could be that students in these 2 studies did not become more knowledgeable with the provided e-learning materials; this may be because the conventional in-class teaching was sufficient or the Web-based materials were not aligned to the assessment outcomes and, therefore, did not support the learning outcomes.

Although most papers reported a positive relationship between more e-learning usage and better academic attainment, there were 2 that reported otherwise. While Boye et al [18] observed a slight increase in students’ grades through the watching of more animated e-learning videos, this was only for students with intermediate scores. Students with either good or weak academic grades did not appear to benefit. They reasoned that students with better academic performance might already be inclined to study more through conventional means, such as books, lectures, or tutorial sessions, limiting any potential additional benefit from supplementary e-learning materials. In contrast, weaker students may try to compensate for their lack of participation during regular teaching sessions by spending more time with the e-learning materials in “the last minute” prior to examinations. In addition, Colthorpe et al [21] showed that better performing students watched lesser videos than inferior students. They reasoned that students with greater understanding of the learning materials may not consume the video content as they have already mastered it. From this, we may infer that watching more videos may not lead to better learning outcomes for particular students, especially if students do not see the purpose or meaning for their learning.

Students’ satisfaction with Web-based learning materials was attributed to its ease of access [22], as well as its usefulness as a “complement” to lectures [32] and a “good supplement” to regular teaching [18]. Students felt that e-learning could increase competence in the subject [13].

Aside from descriptive and diagnostic analytics, higher levels of learning analysis, such as predictive analysis (ie, regression and modeling), could be used to apply learning analytics data within the scope of students’ need. For example, Purdue University’s Signals project is, perhaps, the most famous example of the successful application of learning analytics’ predictive modeling to identify at-risk students. By providing a real-time “red or amber or green traffic light” to students and teachers based on the data collected from the LMS, learning analytics can help in identifying students who are at risk so that help and support could be provided to them [39-42]. Teachers can then target interventions ranging from emailing those who are at risk to referring them to academic advisors or meeting them face-to-face [39]. In another case, the University of Alabama developed a model to predict student retention rate by using freshmen’s Web-based data records with various parameters such as students’ English course grades, total hours earned, and their demographics information [5,43]. These are just a few examples of learning analytics applications in higher education. It is predicted that in the coming years, learning analytics will be widely implemented in Web-based education to identify the patterns of student behaviors for improving students’ learning and their retention rates [5].

### Variations of Learning Analytics Variables

In this review, a diverse range of learning analytics data was reported, making comparisons between the studies at the least difficult. In addition, there was ambiguity with regards to the interpretation and definitions of the learning analytics data. For example, “log-ins” was not clearly defined if it related to the duration of log-ins or the number of successful log-ins [12]. It is important for future studies to standardize and include clear definitions of such key variables to facilitate comparisons.

The most common learning analytics parameter documented was the number of connections to a specific e-learning material. However, some only recorded connections to folders of resources such as webpages containing multiple e-learning resources. For example, Colthorpe et al [21] deposited all of their teaching videos into a single folder for which they registered the frequency of access, and Franson et al [25] tracked students’ access to “TRC database material,” which included schematic graphics, explanation texts, and feedback questions, rather than each type of resource individually. Therefore, we do not have a clear picture of what learning materials students are actually engaging with. It would be preferable to record access to individual e-learning resources, as this would provide more precise data regarding the effectiveness and popularity of each document. Furthermore, this would allow differentiation between accessing e-learning materials as opposed to log-ins for viewing other included documents, such as timetables or course announcements, which would not be considered learning materials.

Another way used to analyze students’ engagement of e-learning was the time they spent on tasks. Nevertheless, the majority of studies, except Boye et al [18] and Franson et al [25], did not provide sufficient details on how this parameter was recorded. Boye et al [18] reported that each student’s individual access to an e-learning animation was recorded at 10-second intervals, and when students had stayed idle for >3 minutes, the tracking mechanism would stop until another action was taken. Franson et al [25] applied filters to time spent on their “TRC database material” such that a too short engagement (3 seconds) was considered not to be meaningful. Likewise, greater than the anticipated duration (6 minutes) suggested nonstudy activities. However, the total time spent on a task may provide limited information, as this may simply be an open webpage not being read or a playing video not being watched. Furthermore, this would neither necessarily reflect the actual physical presence of students nor cognitive engagement.

A total of 8 studies documented the frequency of messages posted in forums. However, the value of such learning analytics data was questionable, as it does not reflect the quality of the discussion students created. As such, unless the quality of forum posts is monitored and assessed, the benefit of simply registering the quantity of posts is in doubt. Therefore, an assessment rubric is required that can evaluate the quality of the discussion posts.

From the above, we can assert that detailing of learning analytics data needs to be improved so that research studies can allow meaningful outcomes and interpretations. Furthermore, diverse analytics need to be recorded for the individual types of e-learning resources, as well as a way of capturing active engagement with the content.

### Recommendations

This review observed diverse approaches in recording and defining learning analytics data of students’ e-learning use. The varied and imprecise nature of learning analytic data that has been used in the current studies does not help further our understanding of how Web-based learning helps students learn and how we can use the analytics to help students at risk. Hence, a more detailed and precise approach to analytics and e-learning research is required to answer these questions.

From an examination of this literature and an analysis of its shortcomings, we propose the following recommendations:

- Learning analytics data should include well-defined terms and conditions used to describe data collected.
- A detailed collection of individual e-learning items should be performed, as opposed to merely platform log-ins or folder connection frequencies to gain greater knowledge regarding learner engagement.
- The length of time spent by students on e-learning materials, should be recorded when appropriate for certain e-learning resources. For instance, the collection of data on the amount of a video watched is now possible with many LMS and even on YouTube. Conversely, time analysis of access to a PDF document would not be appropriate.
- A mechanism to identify idle time in an e-learning activity may be appropriate to identify when students are not engaged with learning by way of analyzing keystrokes, mouse use, or video playback and pause buttons.
- Course designers and researchers need to plan their learning objectives and how these map to in-class and Web-based learning activities. To truly identify the benefits of e-learning, some materials should be exclusively focused on one particular course learning objective, and students should be informed this will not be covered in-class and that this learning objective will be assessed. This will drive consumption of the learning materials and will also allow more meaningful outcome analysis of e-learning to assessment outcomes, as it is not known if in-class learning may be merely duplicating Web-based learning. This requires careful design and planning as to how e-learning is effective and how it helps the course objectives.
- Course designers and researchers also need to consider the best delivery mode of the Web-based learning material, and as of yet research has not answered this question. Ideally, a learning taxonomy needs to be used to classify the nature of the learning objective (ie, understanding, analysis, critical thinking, etc) and this in turn also needs to be mapped to the e-learning material. With such mapping, researchers will be able to build understanding about which knowledge domain is best suited for which e-learning presentation type.
- Indicators as to how cognitively engaged students are with the Web-based learning materials are desirable, as the use of e-learning does not necessarily ensure cognitive engagement. For example, there is a need to test understanding that can be achieved by questions embedded with videos or as standalone assessments to Web-based learning resource; this will help assess understanding of the material as a proxy for engagement.
- Furthermore, course designers may want to look for ways to motivate students to consume the e-learning materials in a more spaced manner rather than at the last moment. Studies have reported that students’ use of e-learning materials were crammed days before exams. Therefore, there may be a need to space smaller assessments over time during the course, which may facilitate consumption and learning.
- Another possible motivator to engage students’ learning with electronic resources may be a personal analytics dashboard to let students see their current activity in e-learning usage and how this compares to others; this may motivate lower consumers to consume more to help keep them on track.
- A particular and significant point on the relation of e-learning analytics to learning outcomes may actually be that all we are doing is measuring the motivation of students and not the benefit of e-learning. Well-motivated students will succeed in virtually any learning environment and, therefore, we may well not be measuring the impact of the e-learning experience. Our efforts should, therefore, be targeted at identifying students who are underperforming in consumption of the e-learning materials and identifying how to motivate or support them. This is the next challenge for researchers.

Table 4 provides a concise table suggesting several points one might consider for example in studying video e-learning through learning analytics.

### Conclusions

This systematic review of e-learning health care education supports the general literature that greater consumption of e-learning as recorded by learning analytics generally supports learning outcomes. However, the detail and nature of the studies were heterogeneous both in learning analytics data and in e-learning content. More detailed and more focused research is required to help understand how e-learning, learning analytics, and learning outcomes can be more effective and in how they help students learning. Recommendations have been proposed for future course designers and researchers to create content and provide evidence for meaningful e-learning and support of all learners as this pedagogical approach grows further.

**Table 4:** An example of learning analytics of a video e-learning study.

Suggestion	Example
Well-defined variables and conditions	Reporting *individual* video access: (a) Number of times video A is watched; (b) Number of times video B is watched; (c) Duration of video A being played; (d) Duration of video B being played Precise *conditions* in recording: (a) <3 minutes between consecutive clicks of videos would not be counted; (b) <5 seconds of duration in playback would not be counted
Exclusive materials	Exclusive learning materials via e-learning (not overlapping with in-class materials) Informing students the e-learning materials would be tested
Mapping learning taxonomy	When creating videos, one may consider classifying into: (a) Video A is for understanding; (b) Video B is for critical thinking
Engaging cognitively	Multiple-choice questions to be embedded within the videos, such that students need to answer them to continue watching
Facilitate consumption and learning	Space smaller assessments over time during the course Personal analytics dashboard showing consumption status and allowing comparisons with the rest of the class

## Abbreviations

LMS: learning management system
MCQ: multiple-choice question
TRC: Teaching Resource Centre
